# Supplementary material for: Acquisition and Retention Effects of Fundamental Movement Skills on Physical Activity and Health-Related Fitness of Children with Autism Spectrum Disorder
Source: Healthcare (Basel). 2024 Jun 29;12(13):1304. doi: 10.3390/healthcare12131304 (PMC11241392; doi:10.3390/healthcare12131304)
Supplement: Supplementary file 1 [file healthcare-12-01304-s001.zip › healthcare-3029926-supplementary.pdf]

Supplementary Material

Acquisition and retention effects of fundamental movement skills on physical activity and health-related fitness of children with ASD

Yu Xing [a, b, *], Haoyan Liu [a], Xueping Wu [c, *]

[a] *School of Physical Education, Hainan University, Haikou, Hainan 570228, China.*

[b] *Hainan Provincial Key Laboratory of Sports and Health Promotion, Hainan Medical University, Haikou, Hainan 571199, China.*

[c] *School of physical education and training, Shanghai University of Sport, Shanghai 200438, China.*

[*] Corresponding author.

E-mail address: xingyu@hainanu.edu.cn, Tel. 18716323578 (Y. Xing).

[*] Corresponding author.

E-mail address: wuxueping@sus.edu.cn, Tel. 15501780712 (X. Wu).

Table S1 Fundamental movement skill intervention content and allotted time for each session of the experimental group

Time	Items	Form	Subject
4 min	Classroom routine	Group	Teachers and students must greet each other in every class, emphasizing “yes”, in any form, such as words, body, eyes, etc.; Joint exercise must be placed after brisk walking, jogging and other activities.
10 min	Warm-up activities	Group	
10 min	Skills review (Line up, walk, run, jump, catch, throw and bat)	Group	Reinforce learned skills or practice different limb functions.
15 min	Social Story skills (Stories about motor skills)	Group/team /individual	Emphasize attention and classroom environment adaptation
15 min	Skill games (Line up, walk, run, jump, catch, throw, bat)	Group/team /individual	The content of each lesson of skill teaching should be progressive and the overall content should also be progressive. Task analysis can be used to decompose the teaching and training content. The teaching of skills can be adjusted based on the child’s performance.
6 min	Relaxation exercises (yoga/meditation) and review of teaching	Group	The parts of the body that are stretched should be the muscles used in the skill and performance area. Children who perform well should be mainly praised during the course and other children should be encouraged to work together. Record what children can and cannot do and how they perform in class. Find out the advantages and disadvantages of teaching and training arrangement.

Table S2 Fundamental movement skill intervention content and allotted time for each session of the control group

Time	Form	Course Content
5 min	Group	Classroom Routines
10 min	Group	Warm-up activities
25 min	Team	Skill Acquisition
20 min	Group/Team	Flag lowering, indoor exercise

Table S3 Content Framework of Skill Intervention in the experimental group

Items	Contents
Lining up	Lining up before class, standing at the point to learn the movement, orderly completing a distance (straight or curved) movement
Walking	Walking in small steps, high legs and around obstacles
Running	Jogging, relaying, running with high legs, running around obstacles
Jumping	Jumping from high places with one or both feet, jumping high, jumping jacks, jumping over obstacles
Catching ball	Catching the ball after leaving the hand, the rebound ball, the ball thrown by the partner, the direction ball, the distance ball
Throwing ball	Two-handed chest passing, dropping the ball (spot, distance), two-handed wall passing, overhand throwing, underhand throwing
Bouncing ball	Clapping the ball with one hand or two hands
Kicking ball	Kicking the ball in place, short distance running with the ball
Others	Original squatting, twisting, cresting expansion.

Table S4 Strategies of how to apply and change the difficulty of content for the Overhand toss

Teaching Strategies	Course instruction	Implementation Strategy
Children's attention	Children arrive at the designed location and the teacher goes through a story or situation that is interesting or relevant to the children's daily lives and embeds the skills learned.	To build children's awareness of rules and improve their attention, through fixed seats, social stories, pictures or video presentations close to daily life to attract children's attention.
Clear and appropriate instructions	Teachers and children work through task cards with clear, easy to understand instructions and intonation.	The teacher demonstrates, cues the key words of the action, repeats the key words during the exercise, and the instruction clear.
A combination of difficult and easy tasks	Maintain skills learning component and improve component 50%.	Provide different difficulty tasks according to children's ability and actual performance, control unarmed to object control, distance, time duration and other difficulty level adjustment.
Share control	Teachers provide opportunities for children to make their own choices.	Children choose the materials they like and choose what tasks to start with
Response to multiple clues	Teacher prompts teaching points through different pictures, videos or stories	Stick pictures to the children's practice area for easy learning
Direct reinforcement of outcome strategies	Provide immediate feedback on the results based on the actual performance of the child	If the child responds correctly, encourage them to continue practicing
Subject to results	Positive feedback is the main motivation for children to reinforce	If children respond correctly, they can be led to participate in their favorite play
Reinforcement attempts	Provide immediate and meaningful feedback based on the child's behavior	Provide immediate and meaningful feedback based on the child's behavior

Table S5 Demographic characteristics of the participants

Category	Experimental group<br>Mean±SD	Control group<br>Mean±SD
Age (y)	8.47±0.21	8.45±0.32
Height (m)	1.36±2.63	1.38±2.72
Weight (kg)	35.27±0.14	36.47±0.25
BMI (kg/m^2)	16.39±4.15	14.64±2.76
Male (n)	9	7
Female (n)	2	3

Abbreviations: BMI, Body Mass Index.

Table S6 Follow-up comparisons on the body composition

Category	Experimental group			Control group		
	Pre-intervention	Post-intervention	Follow-up	Pre-intervention	Post-intervention	Follow-up
BMI	16.46	14.29	15.41	15.90	14.72	15.41
Waist-to-hip ratio	0.91	0.88	0.94	0.88	0.88	0.93
20-meter PACER	3.90	4.73	4.11	3.11	4.10	3.90
Grip strength	4.56	5.73	5.26	4.50	5.32	4.94
Sit-ups	8.34	12.57	14.74	8.34	7.98	8.46
Vertical jump	9.59	10.18	9.76	9.53	9.76	9.57
Sit and reach	7.94	12.88	8.45	7.94	9.15	8.45
